# Katie Erikson's caring theories. Part 2. The theory of caritative caring ethics and the theory of evidence

**DOI:** 10.1111/scs.13098

**Published:** 2022-06-23

**Authors:** Ingegerd Bergbom, Lisbet Nyström, Dagfinn Nåden

**Affiliations:** ^1^ Institute of Health and Care Sciences Sahlgrenska Academy University of Gothenburg Gothenburg Sweden; ^2^ Åbo Akademi University Finland; ^3^ Department of Nursing and Health Promotion Oslo Metropolitan University Oslo Norway

**Keywords:** caring, caritative caring, ethics, ethos, evidence, theory, TheoryCaring science

## Abstract

In this article, Katie Eriksson's theory of caritative caring ethics and the theory of evidence, are described. Both theories are anchored in caritas, that is love, mercy and compassion. The theory of caritative caring ethics was first described by Eriksson in 1995, where seven assumptions or basic categories were elaborated. These were: the human being's dignity, the care relationship, invitation, responsibility, virtue, obligation or duty, and good and evil. Eriksson's theoretical contribution is that she makes a distinction between caring and nursing ethics, between inner and external ethics, and between natural and clinical ethics. Concerning the theory of evidence, Eriksson claims that a multidimensional scientific view of evidence in caring that focuses on the patient's world is necessary and vital. To see, realise, know, attest and revise constitute the ontological definitions of the concepts of evidence and evident. The theories are united by the core concepts of testimony and witnessing the human being's suffering. Eriksson points out that it is in the ethical acts that deeds are formed, based on ethos. The anchorage in an ethos means to have firm value‐loaded judgements of an inner motive. Moreover, the anchorage in ethos presupposes a personal and natural ethic. The good deeds are realised in the relationship between the patient and the carer, but the caring ethics is not a professional or external ethics. Caring ethics is an ontological inner ethics meaning fellowship and the right to exist, but it is the patient's world and reality that decides the foundation and starting point for caritative caring ethics in clinical practice. The ultimate purpose and goal of caring are to guarantee the patient's dignity and absolute value as a human being.

## INTRODUCTION

This article aims to describe Katie Eriksson's theory of caritative caring ethics and the theory of evidence. Both these theories have their roots in caritas. Caritas, that is mercy and love, is the foundation and principle for uniting different fragments of knowledge into meaningful wholeness. This foundation is valid for all Eriksson's caring theories [1] and thus also for the theory of caritative caring ethics and the evidence theory. The theory of caritative caring ethics was first described by Eriksson in the research report “Towards a caritative care ethics” [2], in 1995. At first, her focus was on morals and ethics but during the following years, she came to focus mainly on ethics and ethos [3, 4]. Eriksson [2] makes a distinction between caring ethics and nursing ethics, as well as between inner and external ethics and between natural and clinical ethics. Inner ethics is ontological and is about what a human being is and what happens in relationships among human beings. It is about being good and doing good. External ethics is about ethical guidelines, about what is the right thing to do, according to ethical principles and rules. Nursing ethics deals with ethics of justice. Eriksson [2], inspired by Nygren's human ethics [5] and Levinas [6] “face ethics”, states that caring ethics is an inner ethic and a natural ethic. She [7] also emphasises that caring ethics, as clinical, has different characteristics within different contexts, but its focus is always the personal encounter between the patient and the carer in a caring relationship. A basic thought and starting point in the theory is the supposition of human holiness and absolute dignity. The theory of caritative caring ethics is closely connected to evidence theory by its anchor in caritas, which means love, mercy and compassion.

Eriksson [ 8, p. 362] refers to Schopenhauer [9] who thinks that compassion is the original phenomenon of ethics. Moreover, another concept and phenomenon that connects the theory of caritative caring ethics and the theory of evidence is testimony. Testimony is understood as a core concept and concerns ethical responsibility and the question of seeing or not seeing a human being's suffering [10].

## THE THEORY OF CARITATIVE CARING ETHICS

In the research report “Towards a caritative care ethics” [2] Eriksson describes seven basic categories of care ethics, fundamental to the theory of caritative caring ethics inspired by Nygren's [5] thoughts about ethical disposition. The basic categories are: the human being's dignity, the care relationship, invitation, responsibility, virtue, obligation or duty and finally good and evil. The ultimate purpose of caring ethics is to safeguard the human being's dignity and value as a human being, and the right to self‐determination. Therefore, the care relationship is of vital importance concerning the carer's motive to care, responsibility, ability to be compassionate, and will to invite the patient into a relationship. Eriksson [2] talks about the absolute dignity, i.e., the first category, which means a right to be confirmed as a unique human being.

Human dignity is the most fundamental value of the caring spirit. Human dignity is partly absolute dignity and partly relative dignity. Relative dignity is influenced and formed through culture and external contexts [11]. The absolute dignity of man is that man is a human being. In this respect, all people are equal and inviolable. Eriksson refers to Pico who states that man's freedom is the basis of man's dignity [12]. The author also refers to Lindborg [13] who argues that man himself has an ability to shape his life and his being. Man's absolute dignity means holding the human office, to serve in love, to exist for the other [14, 15]. This basic ontological assumption was later formulated as follows: man is basically holy.

In the biblical sense, man has gained dignity by being created in the image of God. Man experiences absolute dignity when fulfilling man's task as a human being, i.e., serving and existing for another human being. When a person is deprived of responsibility, the person is at the same time deprived of dignity. Human dignity even includes the freedom to make one's own choices in life, and the right to protect oneself from intrusion [12].

Absolute human dignity is grounded in one's humanity [16]. The deepest ethical motive in all caring involves respect for the absolute dignity of the human being. Human dignity implies inner freedom and responsibility for one's own and others' lives [17]. Eriksson states that care that is thoroughly ethical distances itself from all forms of condemnation, the exercise of power, punishment and various forms of non‐care [12, 16]. A person can violate his own dignity by not being the person that deep down he wants to be [ 12, p. 82]. Eriksson refers to Levinas [6], who believes that dignity is to see the other as the other person is and to bear responsibility for this person. Eriksson continues to state that to really see the patient, is to take the patient seriously, not questioning or exposing the patient. This means taking care of the patient in the deepest sense [12]. Human dignity is the hub around which ethical thinking revolves and it shapes the ‘Gordian knot’ of ethics which constantly tends to be broken (Eriksson [ 8, p. 15].

The second category is that ethics is relational, because it is in relationships that the ethical appears. It is in the encounter that ethics becomes visible and real. Eriksson [2] refers to Levinas [6] and the responsibility for the other when seeing the other's face. It is not enough to have knowledge in ethics; it has to be integrated in the person and made visible in the person's action or activity. The meaning and the synonyms of the concept of the relationship are narrative, relation, connection and touch [18]. All these synonyms and their meanings have an ethic potential, according to Eriksson [2]. For example, connection is closely connected to obligation, promise and duty. To promise or to undertake an obligation means to be prepared to sacrifice something. Eriksson [2] claims that an ethical care relationship has to be caritative based on love, attention and a will to listen to and share the other person's story, suffering and “life circumstances”. For this to happen there must be a will to be connected and touched.

The third category is invitation, where Eriksson [2] states that the foundation of ethical care is how we can invite the other person and offer a caring relationship. Eriksson uses the word “guest of honor” because this term includes all ethical categories such as dignity, responsibility, respect. The invitation also means to welcome the other person into a relationship, and this invitation is not demanding. Eriksson [2] here refers to Kierkegaard [19] who, in turn, refers to the Bible and the possibility to find rest, protection and peace. Such invitation and welcoming are based on love and not demand. The concept “invite” and its content was investigated, using a hermeneutic concept determination, by Portaankorva et al. [20]. The result of the etymological and semantic analysis based on Swedish dictionaries of the concept of invitation showed the following dimensions of meaning: openness and a supporting, playful and enthusiastic invitation without expecting something in return [20]. A vital force within invitation is the caring ethos, the ethos of love and mercy. Without this caring ethos, invitation is not welcoming; instead it contains loveless, egoistic and task‐oriented performance without caring. Based on the ethos of love and mercy the invitation is a caring relationship and caring communion.

Eriksson [2] claims that ethics is responsibility, which is the fourth category. Responsibility occurs when we look at the other person's face, and Eriksson here refers to Levinas [6]. Responsibility can be seen as an inner, deep personal standpoint or as an outer responsibility, a duty based on ethical rules or codes. This latter responsibility can be performed, without any personal engagement, while the inner responsibility demands a relation where an encounter between human beings will be established and where both parties are prepared to sacrifice something of themselves.

Responsibility as relation and obligation presumes a relationship, where “the promise” is included. The content of the promise is an engagement to something we are responsible for and that we find meaningful [2]. However, responsibility is also connected to answering, duty and guilt/liability, and by this Eriksson [2] means a commitment and an obligation. Moreover, guilt is tied to love, kindness, and a wish to do or help another person. Thus, responsibility is based on caritas and compassion, as compassion is the core of ethics. Eriksson claims that feeling compassion is understood as an ethical act and is not the same as feeling sorry for another person. Love as an ethical act has been further elaborated in an article by Thorkildsen et al. [21]. The authors described that love was expressed by tone, face, eyes, hands, and the form of words. Love is seen as a holy power, and it is in the encounters when we face the suffering human being's needs and desires that love becomes a power. Love is visible and expressed in the carer's actions, and love has to be demonstrated in practice such as in spontaneity and engagement. Moreover, love as an ethical act appears in behaviour and attitude.

Eriksson [2] concludes that human beings are prepared and want responsibility, which is connected to dignity, reverence and devotion. Eriksson’s thoughts presented in 1995 [2] and 2003 [7] were further discussed and developed by Wallinvirta [22]. In the semantic analysis of the concept of responsibility, the following dimensions were found: responsibility as obligation and liability, duty and relation, responsibility as answer and guilt/liabilities and punishment. Based on the semantic and discrimination analysis, Wallinvirta [22, p. 181] summarises the findings from these two analyses in four categories of responsibility: (1) as condition and borders/limits, (2) as imperfection and infinity, (3) as “bound to” and relation/connection, and (4) as action and judgement. In the light of caritas, Wallinvirta [22] found that responsibility is connected to freedom, obligation and love, which exist in a tension between an inner and an outward (outer) ethics.

The fifth category that Eriksson [2] described is the statement that ethics is virtue. A basic condition for the caritative ethics is the ability to embrace the disposition and attitude of love and the spontaneous mercy and obligation. Eriksson uses the words “unconditional action” in caring, which is founded in the will to do good. This unconditional act is something inside each human being and it cannot be mediated by knowledge. A condition for being spontaneous and unconditional in caring actions is having experiences of freedom, courage and consideration. Ethics as virtue has been further researched and elaborated by Näsman [23] and Näsman et al. [24] by using concept analysis. They found, by an etymological investigation, that virtue is about power or strength, goodness and art. In their semantic analysis, three dimensions were identified: (1) Virtue causes something to be well functioning, (2) Virtue makes the human being good, and (3) Virtue gives the human being morality and decency. This means that virtue is a power that makes it possible to realise the good in relation to the other human being/patient. However, this assumes that virtue is anchored in ethos that connects the dimensions of virtue as goodness with power and art. Thus, virtue can be understood as a power that makes it possible to realise that which is good for human beings [23, 24]. Ljungquist [25] found that caritative care ethics means good habits that can alleviate suffering and promote health, and all forms of caring deeds and good habits are connected to the virtues and moral integrity of the caregiver.

The sixth category is the statement that ethics is duty or obligation, which is seen as a necessity including a promise. Eriksson [2] described duty and obligation as external or inner, an unconditional relation, a promise that is experienced as an action of love. Eriksson refers to Kierkegaard [26] who also sees duty and obligation as an inner relationship. Duty and obligation is something that rests upon us as human beings. In connection with this thought, Kierkegaard has the opinion that it is each human's duty to have a mission. It is vital for human beings to have virtues because these virtues are conditions for unconditional, spontaneous and voluntary obligation.

The last category is the good and the evil [2]. Based on the thought that humans have free will, Eriksson stated that humans can choose to do “good” instead of evil. In caring, this means that the unconditional actions are founded in the carer's will to do “good” for the patient, and this is something inward and in one's heart, which cannot be mediated through knowledge. Guilt is described and understood as existential, and Eriksson here refers to Buber [27] and Jaspers [28]. Conscience is connected with our experience of existential guilt. When we insult something that is felt to be fundamental to our existence in the world, we feel guilt, an existential guilt. It is our conscience that prevents us from doing evil. Eriksson [7] points out that it is in ethical acts that good deeds belong, and that ethos is the foundation for how these deeds are formed. The anchorage in ethos creates necessary conditions for a personal inner ethics, including a will to do the good, the true and the beautiful [24]. The good deeds are realised in the relationship between the patient and the carer, but the caring ethics is not a professional or external ethics. Caring ethics is an ontological inner ethics that gives space for fellowship and the right to exist, but it is the patient's world and reality that decides the foundation and starting point for caritative caring ethics in clinical practice. The main purpose and goal are to guarantee patients' dignity and absolute value as a human being.

From the year 2000 and during the following years, Eriksson further developed her thoughts about caritative ethics and the need for good deeds. The foundation for ethical deeds is the idea about freedom to act. About this idea, Eriksson [7] refers to Piltz [29] and the idea of the human being as an image of God. Freedom to act is understood as a result of a dialogue between common sense and will, but the unconditional and absolute source for the act of will presupposes fidelity to oneself. The human being is forced to choose between good and evil, and this is obvious in words and deeds.

### Ethos and ethics

The basic order of principles in caring is constituted of ethos, caritas, the coherence of meaning and the caring communion, where ethos reflects the basic values in caring such as human dignity and inviolability, the holiness of life, and caritas [30, 31]. The concept of ethos comes from the Greek language and is associated with morals, habits and character, but also “home”. In the book Vårdvetenskapliga begrepp/Concepts in Caring Science, Eriksson and Bergbom [32] state that the concept of ethos contains ethical and aesthetical nuances. These two nuances constitute the melody and tune and “the spirit in the house”, that creates cosiness, comfort, and a feeling of fellowship and belonging. To assent to ethos means to be keenly alive to one's own heart [7]. The meaning of ethos as home is the feeling of being safe, protected, and able to find rest and peace. In a deeper meaning, it also means courage to be the person one wants to be. Ethos, dignity and arête are closely linked. Eriksson claims that a person who has acquired an ethos is at home in his own life and radiates love. This human being is the bearer of natural dignity, has freedom, and is responsible and in the service of man. The person has an arête, i.e., a desire to do the best [ 8, p. 9].

It is ethos that makes us strive to understand the other human being and gives us the insight that each human being is a secret script. This secret script is about holiness and absolute dignity. In caring, this means that we have to practice openness, wakefulness and reverence. Moreover, ethos brings substance to words that express the caring mission and identity, and thus these words will mediate the good, beautiful and the truth. When ethos is incorporated into clinical praxis, it means to take responsibility for patients' dignity so that good caring becomes evident. Von Post [33] describes dignity as the ethos of professional natural care, which makes the carer obliged to protect the patient's dignity and alleviate suffering. It is our conscience and our feelings of guilt that evoke responsibility.

The core of ethos in caring and caring science is caritas, as mentioned earlier, but Eriksson [7] states that this is also an important theoretical perspective when we form our views, in research as well as in practice in order to realise the true, the beautiful and the good. Eriksson [7] describes the concept of ethos, and she concludes that ethos reflects the actual rank of values, it is a tune and a melody that is open to the eternal and the holy. Ethos is a bearer of fellowship and communion, and it refers to “home” in the meaning of protection and rest. To affirm ethos is to listen to the voice of one's heart. Moreover, Eriksson [7] states that it is through caritas that the idea of mercy and love in caring becomes meaningful.

Ethos and ethics are related/connected, as ethos provides ethics with a foundation of values. Ethos is also an attitude including responsibility and human beings' decisions and choices. From ethos, ethics and ethical actions are formed and the ethics of caring is a kind of ontological ethics. Eriksson [7] claims that ethos is an inner appeal of “what we ought”, a caring ethics which has its own language and tone. The inner logic in the ontology of caring is formed from an ethical vision about good caring, but the ethics of “ought” is not a coercion; instead it is a mission of love.

Based on these thoughts, Eriksson [7] thinks that “theoria” (cultivation and character‐building) becomes ethos by being called to serve a certain task or mission – a mission to perform actions that are caring. Moreover, Eriksson [7] states that when theoria becomes ethos it means that the theory becomes real in an ethos, a basic value, which is visible in a person's actions, attitudes and character. By this argument, Eriksson [7] concludes that theoria is praxis.

Another important issue in the caritative caring ethics is the idea that ethos encourages us to see each human being, in a symbolical meaning, as a secret text that has to be learned, read and interpreted. The foundation of ethical actions is the idea of freedom to act and free will. The act of will has its roots in the unconditional, which imply loyalty to oneself and consciousness about good as well as evil. Ethos and ethics teach us to be alert.

Eriksson [7] also states that *ethos is the core of culture* as it is seen as the basic force within a culture. Ethos is beyond the individual as it is common to all. The caring culture is based upon reverence for human beings and their dignity and holiness. The inner rank of values and thus the ethos and culture are changing, and values are changing, but the original ethos may always be evoked, independently of cultural changes. In the description of the concept of “culture”, three central dimensions were elaborated: respect, nurse/care and cultivation. These concepts are important as they connect ethics and ethos with the core concepts in the caritative caring theory. The basic motives to preserve and develop a culture exist in the human being's will to respect, nurse, care and cultivate our culture and our existence on the planet Earth. Concerning this issue, Eriksson [7] refers to the Bible where human beings are entrusted to care for and nurse the earth and all living creatures and plants. Eriksson [7] concludes that cultures are developed in the interplay between human beings, and a culture can be healing as well as divided, good or evil.

A central statement by Eriksson [7] is the *ethics of Words and the living Word*. The starting point is the assumption that words and language are fundamental to a human's being in the world as each person bears words and texts that means something for the individual. The words, the text and the language form the human being. When we articulate the words, our being and ethos are mediated/brought about. The words mediate something of the person's inner world and being. Thus, ethics become concrete in the words and in our language. Speech or logos (word and meaning) characterise human beings as living beings and create our reality. Words are necessary for the creation of fellowship, and words are life. Words form the human being's texts and stories, and these texts and stories open possibilities for understanding the individual human being's world. Moreover, the choice of words is vital concerning the ability to bring the caring cultural heritage to the next and coming generations. According to Eriksson [7], synonyms of the word “word” are promise, pledge, commandment and honour. Eriksson concludes that words create our reality.

Ethics unite tradition and vision. Eriksson [7] has the opinion that our inner wealth is our culture and history as it constitutes the core of our personal values, and ethos unites the inner and outer reality. Caring has a long tradition and development, characterised by an ethos of compassion and love which is fundamental to humans and their lives, and this ethos will endure and survive [17].

## THE THEORY OF EVIDENCE

The theory about evidence has its roots in the ethos of caring, in caritas that is love and mercy [10], and the human being's dignity. The core and idea of caring is caritas, which is the original, natural and the truthful. Eriksson's thoughts about evidence‐based care were first presented in the report “The Trojan Horse” [34], and further developed in 2004 [35]), 2009 [36, 37] and 2010 [10]. In this article a brief presentation is made.

According to the above‐mentioned authors' opinions, the discussion about evidence‐based care has been dominated by a medical technical idea and an emphasis on profession and method, where the question about the caring substance is forgotten. The discussion about evidence has mainly focussed on the use of results from research in praxis, and has lacked a patient perspective and a multidimensional scientific view. The authors [35] argue for a return to the original, historical and semantic emphasis and signification of evidence. They also state that a caring perspective on the concept of evidence that contributes to a greater knowledge and understanding of caring and the patient's world is important. As a starting point, Eriksson [10] describes and explores the concepts of evidence and evident; to see, to realise, to know, to attest and to revise constituting the ontological definition of the concepts.

The concept of evidence in caring science is anchored in the human science tradition, and contains an ontological and contextual dimension. Eriksson [37] suggests a return to the original or ontological evidence model and to the ideas about the truth, the beauty and the good. Such evidence means that the core and substance of caritative caring becomes visible in thoughts, words, actions and in deportment and attitudes. By contextual evidence, Eriksson [10, 37] means such issues that are visible in practice, in caring situations and context, where a human being is involved. Eriksson refers to Martinsen [37] and her thoughts about the patient's world and the fundamentals in life such as joy, love, grief and pain. Furthermore, Eriksson [37] has the opinion that it is easy to disregard these phenomena even if we know that joy, love, grief and pain are important feelings and experiences in the patient's world. However, knowing the patient's world is vital for the carer's ability to alleviate suffering and to promote, protect and preserve health and life.

Evidence means something that is obvious, distinct, indisputable and visible. The word evidence is related to “know” which means to feel, experience, have insight and see in the sense of to examine and review. Etymologically, the concept of evidence also means attestation or witnessing, revision and vision. By witnessing, it is possible to make something visible, and at the same time the responsibility becomes present. In this regard, Eriksson [37] mentions King's evidence, which refers to power and truth.

Eriksson and Nordman [35] claim that evidence cannot be obtained only by scientific knowledge and research results. Instead, Eriksson claims that it is obtained by a unification of science, art and ethics. Science refers to the head, art to the hand, and ethics to the heart. The original concept of evidence is connected to the head‐hand‐heart thought, where head symbolises seeking for the truthful, the hand symbolises the art of caring, the esthetical and beautiful, and the heart represents the ethical and the good. Thus, evidence and ethics are connected, but they are also connected to Claritas [38], the light that is necessary for insight and seeing. Claritas might facilitate caritas in carrying out what is good in a responsible, courageous and Âretian way, which means that all caring is grounded in ethos and wisdom [39].

According to Eriksson and Nordman [35], the theory of evidence is a dialectic movement between ideal and reality. To be able to seek and gain knowledge about the substance of caring and be able to make this substance visible, there must be a vision about the good care/caring. Both proof and testimony are required to provide evidence. To revise means that based on the evidence and testimony, changes and renewal of the care practice are possible. It is by evidence that arguments for true scientific knowledge can be conveyed. By witnessing there is a responsibility to attest what has been seen and known. Witnessing includes an ethical aspect, which demands expression in words [40]. The best evidence needs both proof and testimony [35].

## CONCLUSION

The theory of caritative caring ethics and the theory of evidence have their roots in the theory of caritative caring and thus in love, mercy and compassion. Both theories have been developed and revised over a period of 25 years. Eriksson states that the creation of theories starts with observation in reality, and these theories can be based on assumptions or hypotheses that can be validated, further investigated and developed. Eriksson developed the theory of caritative caring ethics based on seven assumptions. These assumptions were based on literature and etymological and semantic analysis of concepts and their relations. Eriksson's theory of evidence is based on a multidimensional concept of evidence that includes both an inner ontological and an external contextual dimension. Evident means that which is truthful, good and beautiful. Moreover, the theories are closely connected to each other by ethos, by a unification of the logic of the head, hand and heart. Eriksson et al., conclude that ethics and evidence that are not founded in ethos lack the idea of “good caring” [34].

## AUTHOR CONTRIBUTIONS

Made substantial contributions to design: IB, LN, DN Drafting the first version of the manuscript: IB Revising the manuscript for important intellectual content: IB, LN, DN Given final approval of the version to be published: IB, LN, DN Agreed to be accountable for all aspects of the work in ensuring that questions related to the accuracy or integrity of any part of the work are appropriately investigated and resolved: IB, LN, DN.

## ETHICAL APPROVAL

Not applicable.

## CONFLICT OF INTEREST

None.
